# Association between the lunar cycle and pregnancy at first artificial insemination of Holstein cows

**DOI:** 10.3168/jdsc.2024-0722

**Published:** 2025-03-04

**Authors:** P. Pinedo, K. Keller, M. Schatte, J. Velez, T. Grandin

**Affiliations:** 1 Department of Animal Sciences, Colorado State University, Fort Collins, CO 80523; 2 Department of Statistics, Colorado State University, Fort Collins, CO 80524; 3 Aurora Organic Farms, Platteville, CO 80651

## Abstract

The objective of this observational study was to analyze the association of the lunar cycle with pregnancy at first artificial insemination (P/AI1) of Holstein cows. The study cows were managed under organic certification, which prohibits the use of exogenous hormones for synchronization of ovulation and requires continuous access to open dry lots. Two categorizations of the lunar cycle were used for the analysis: lunar phases 4 (LP4) with 4 categories of equal duration (new moon to first quarter, first quarter to full moon, full moon to third quarter; and third quarter to new moon) and lunar phases 8 (LP8) with 8 categories (new moon, first quarter, full moon, third quarter [set to 1 d duration each] and waxing crescent, waxing gibbous, waning gibbous, and waning crescent [4 d of duration each]). Two sets of data were created where first postpartum inseminations were matched with the corresponding lunar phase for the 2 categorizations (LP4 and LP8) using the artificial insemination (AI) dates. Data were examined separately in primiparous and multiparous cows. Least squares means for P/AI1 by lunar phase were calculated and compared using ANOVA. Potential associations between P/AI1 and the 2 lunar phase categorizations were initially tested by chi-squared test of independence followed by multivariable logistic regression. Differences in P/AI1 LSM were established only in primiparous cows in the LP8 categorization. Cows receiving AI1 during the first quarter had a smaller probability of P/AI1 (0.29) as compared with waxing gibbous (0.39), waning crescent (0.40), waning gibbous (0.42), and third quarter (0.46). The logistic regression analyses were completed using the new moon category as reference. The analysis identified greater odds of P/AI1 during the third quarter than during the new moon in both primiparous (odds ratio [95% CI] = 1.15 [1.02–1.30]) and multiparous (1.12 [1.01–1.24]) cows. When LP8 was considered for the logistic regression analysis, associations between the lunar cycle and P/AI1 were only identified in primiparous cows for the first quarter compared with the new moon (odds ratio [95% CI] = 0.67 [0.47–0.94]). In conclusion, this study identified small associations between the lunar cycle and pregnancy at first insemination of Holstein cows maintained in an organic-certified herd. Validating the associations described here and clarifying the biological basis of the reported differences in P/AI1 would require controlled studies in other cattle populations.

## Highlights

• Some evidence indicates that the moon affects specific aspects of reproductive activity.
• Cows had constant outdoor access and were not subject to hormonal reproductive interventions.
• We identified small associations between the lunar cycle and P/AI1.

## Graphical Abstract

**Figure:**
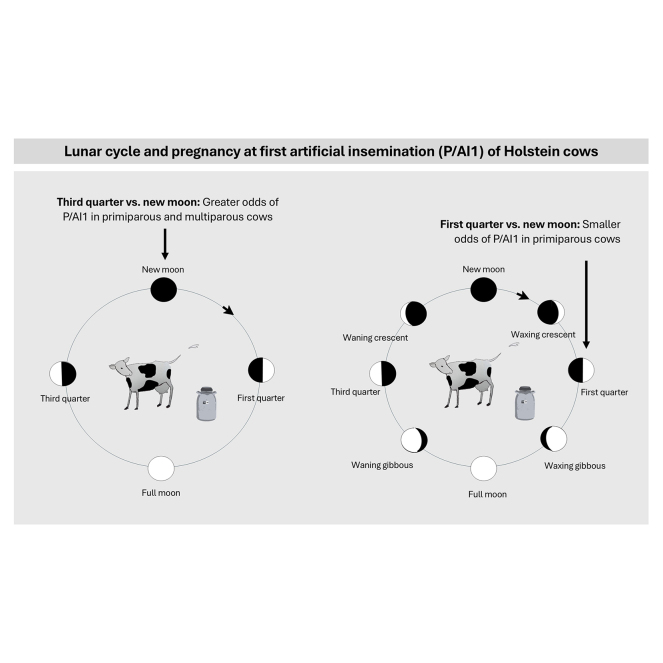
**Summary:** There is a popular belief that the moon phases influence animal behavior and some physiological aspects of reproduction. Our objective was to analyze the association of the lunar cycle, divided into 4 and 8 phases, with pregnancy at first artificial insemination (P/AI1) of Holstein cows managed under organic certification. Differences in P/AI1 were established in primiparous cows when the lunar phases were categorized in 8 periods, with cows that received artificial insemination during the first quarter having the smallest probability of being pregnant. Moreover, the logistic regression analysis identified associations between the lunar phases and pregnancy in both the 4- and 8-phase categorizations. In conclusion, this study determined small associations between the lunar cycle and P/AI1 of Holstein cows maintained in an organic-certified herd.

The influence of the moon on various biological functions in animals and humans has been a subject of interest (Zimecki, 2006; Chakraborty, 2014; Andreatta and Tessmar-Raible, 2020). Some evidence indicates that the moon affects specific aspects of the reproductive activity of multiple species (Canivenc and Bonnin, 1979; Yonezawa et al., 2016; Raible et al., 2017). In humans, most of the research completed in this area relates to the effect of the lunar cycle on synchrony of menstruation and the onset of parturition, although the results are ambiguous (Martens et al., 1988; Arliss et al., 2005). Nonetheless, while several studies have failed in detecting associations between the ovulation time and the lunar cycle (Foster and Roenneberg, 2008), some reports claim synchrony between women's reproductive cyclicity and luminescence or gravimetric cycles of the moon (Cutler, 1980; Helfrich-Förster et al., 2021).

Although scarce, some reports on farm animals have described the influence of the lunar cycle on some reproductive events. In a study completed in dairy cows, Yonezawa et al. (2016) described a significant relationship between the distribution of spontaneous deliveries and specific lunar phases among multiparous animals. Interestingly, in a more recent study, Perea et al. (2024a) also reported an effect of the lunar cycle on calving distribution, gestation length, and calf birth weight. Related to fertility, Kollerstrom and Power (2000) indicated that the conception at first mating in mares increased progressively from the first quarter and reached the highest value shortly after the full moon. In an earlier report by Horák and Potucek (1978), an effect of the moon on the onset of cyclicity of growing ewes, as well as on conception rate, number of lambs, and fertility was reported, while Palacios and Abecia (2014) determined that the number of lambs obtained per inseminated ewe was higher during the new and decrescent moons.

In cattle, Popescu et al. (2017) reported that breedings during the full moon phase had the highest conception rate in Holstein, Angus, and Limousin cows. Furthermore, Aguirre et al. (2021) reported that the lunar cycle significantly influenced first service conception and frequencies of calving and estrus in tropical Brahman crossbred cows managed in a pasture-based system. Finally, in a recent study by Perea et al. (2024), pregnancy rates at 30 and 60 d for in vitro-produced embryos from oocytes collected from Gyr donors were greater in the waning (from full to new moon) than in the crescent (from new to full moon) phase of the lunar cycle. Notably, pregnancy rate was similarly influenced by the lunar cycle on both the day of the transfer and the day the oocytes were collected.

The presence of multiple external factors, such as the use of synchronization of ovulation and permanent confinement, makes it difficult to analyze the potential associations between the lunar cycle and the fertility in modern dairy cattle. As the use of hormones (excluding oxytocin for postpartum disorders) is prohibited in organic-certified dairy production in the United States and continuous access to an outdoor dry lot is required by organic regulations, these farms provide an opportunity for the analysis of the influence of the moon on various biological functions, including reproduction.

In this study, we hypothesized that conception at first service would vary depending on the lunar phase when the cow was artificially inseminated. Therefore, the objective was to analyze the association of lunar cycle with pregnancy at first artificial insemination (**P/AI1**) of Holstein cows bred based on artificial insemination (**AI**) using estrus detection through activity monitoring with no use of exogenous hormones to manage reproduction.

This observational study used retrospective data from a commercial dairy and the research procedures were approved by the Colorado State University Institutional Animal Care and Use Committee Waiver Subcommittee (protocol #6321). The research was conducted in a single organic-certified dairy herd located in Northern Colorado. The analysis included information from 24,983 first postpartum AI from January 1, 2019, to December 31, 2021, in 13,558 Holstein cows.

Cows were housed in barns with 200 to 250 cow capacity freestall pens, provided with sand bedding, headlocks (75 cm of feed bunk space/cow), and continuous access to an outdoor dry lot and to ad libitum water. The stocking rate in the fresh pens was 80% and this rate was maintained around 100% in the subsequent groups. Freestall cleaning and manure removal from the barn's alleys were performed daily during each milking, and scraping of the dry lots was completed every other day. Lights located on the roof of the barns were maintained on during the night and turned off during the day. The dry lots remained open throughout the year. Under extreme weather conditions during the winter, access to the dry lots could be temporarily restricted.

The grazing season extended for at least 120 d/yr, from April 26 to September 30, April 21 to September 26, and April 24 to September 30 in 2019, 2020, and 2021, respectively. During the grazing season, the study cows obtained at least 30% of their DMI from pasture. In this period, cows were housed temporarily in the freestall barns, where the accompanying TMR was provided. The TMR was based on corn silage; wheat silage; grain mix containing soybeans, soy hulls, corn, wheat, and minerals and vitamins; sorghum silage; alfalfa hay; grass hay; and pasture grazing (estimated as 30% to 38%). Grazing management considered rotational grazing in pastures based on perennial alfalfa, annual ryegrass, Italian ryegrass, teff grass, oat ryegrass, sorghum pasture, and native grass pasture. If the maximum temperature was above 29.4°C, cows were moved out for grazing during the night.

Cows were milked 3 times/d depending on their milk yield and a TMR was fed twice daily to meet or exceed the nutritional requirements for lactating Holstein cows producing 30 kg/d milk (3.5% fat and 3.1% true protein; NRC, 2001). After 45 DIM, all of the cows were moved into insemination groups and subject to AI throughout the whole year. No exogenous hormones were used to manage reproduction, and cows were bred based on AI using estrus detection through continuous activity monitoring with an IceQube 3-axis accelerometer attached to the cow's rear leg (Peacock Technology Ltd., Stirling, Scotland). An algorithm considering multiple activity variables and cow reproductive information predicted the occurrence of estrus and generated a daily list of cows to be bred. If estrus was detected, cows were inseminated during the morning. Pregnancy diagnosis was performed by the attending veterinarian by transrectal palpation of the uterus and its contents at 45 ± 7 d after AI. To be included in the analysis, each AI needed to either have a subsequent pregnancy diagnosis or undergo a second AI within 30 d. If a second AI was performed, the first AI was deemed unsuccessful in establishing a pregnancy.

Two categorizations of the phases of the lunar cycle were considered for the analysis. The initial categorization included 4 lunar phases (**LP4**) of equal duration: new moon to first quarter, first quarter to full moon, full moon to third quarter; and third quarter to new moon. The second categorization included 8 lunar phases (**LP8**). To better isolate the potential effect of the 4 main lunar phases (new moon, first quarter, full moon, and third quarter), their durations were set to 1 d duration for the LP8 categorization. Dates for these 4 phases were selected considering the day of 0%, 50% 100%, and 50% moon visibility, respectively. Next, the dates of the previous and subsequent day to each of these 4 phases were removed from the dataset. The 4 periods between these specific lunar phases had 4 d of duration and included waxing crescent, waxing gibbous, waning gibbous, and waning crescent. Finally, 2 sets of data were created where each AI was matched with the corresponding lunar phase for the 2 categorizations (LP4 and LP8) using the AI1 dates. Artificial inseminations occurring during the days that were excluded from the LP8 categorization were removed from the LP8 analysis.

Statistical analyses were completed using SAS 9.4 (SAS institute Inc., Cary, NC). Descriptive statistics for the variables of interest were calculated using the PROC FREQ and PROC UNIVARIATE. Data were examined separately in primiparous and multiparous cows. Least squares means for P/AI1 by LP4 and LP8 category were calculated and compared using ANOVA (PROC MIXED). Potential associations among pregnancy at AI1 and both season and lunar cycle were initially tested using the chi-squared test of independence (PROC FREQ). Subsequently, multivariable models were tested by logistic regression using PROC GLIMMIX. The main explanatory variable in the ANOVA was lunar cycle category (LP4 or LP8) and the covariables used in the models included season of AI and DIM at AI. Cow ID was added as a random effect into the models to account for repeated measures on cows across years.

The logistic equation to investigate the effects of lunar phases can be expressed as presented by de Mutsert et al. (2009):

ln [*p*/(1 − *p*)] = β_0_ + β_1_(lunar phase) + β_2_(COV) + β_3_(lunar phase × COV),

where ln is the natural logarithm, *p* is the proportion of cows diagnosed pregnant after AI1 and [*p*/(1 − *p*)] are the odds of this outcome, β_0_ is the model intercept for the study outcome, β_1_, β_2_, and β_3_ are the regression parameters for lunar phase category (LP4 or LP8), the proposed covariables (COV; season of AI and DIM at AI), and the interaction term lunar phase category × COV.

Interaction terms remained in the models at *P*-value ≤ 0.10. Statistical significance was established at *P* < 0.05 level using a likelihood ratio test.

The distribution of AI1 (n = 24,983) by season was 30.8%, 17.5%, 25.1%, and 26.6% for fall, winter, spring, and summer, respectively. Overall, for the 4 lunar phases categorization the distribution of AI was 24.9% for new moon, 24.9% for first quarter, 25.0% for full moon, and 25.2% for third quarter. For the 8 lunar phases categorization the distribution of AI (n = 18.222) was 4.88% (new moon), 19.8% (waxing crescent), 4.83% (first quarter), 19.9% (waxing gibbous), 4.55% (full moon), 20.6% (waning gibbous), 4.98% (third quarter), and 20.5% (waning crescent).

Pregnancy per AI1 were 39.1% and 29.6% in primiparous and multiparous cows, respectively. In primiparous cows, P/AI1 by season of AI were 39.6%, 46.5%, 38.6%, and 32.7% for fall, winter, spring, and summer, respectively (*P* < 0.001). In multiparous cows, P/AI1 by season of AI were 29.2%, 38.6%, 23.8%, and 22.0% for fall, winter, spring, and summer, respectively (*P* < 0.001).

The predicted probabilities for P/AI1 by lunar phase category are presented in Figure 1. The *P*-values for the main effect of the variable lunar cycle in primiparous and multiparous in LP4 were 0.006 and 0.07, respectively. Values in primiparous and multiparous in LP8 were <0.001 and 0.31, respectively. Differences in P/AI1 LSM were established only in primiparous cows in the LP8 categorization, with no differences identified in the LP4 categorization. Cows receiving AI1 during the first quarter had a smaller probability of P/AI1 (0.29) as compared with waxing gibbous (0.39), waning gibbous (0.42), third quarter (0.46), and waning crescent (0.40).

**Figure 1:**
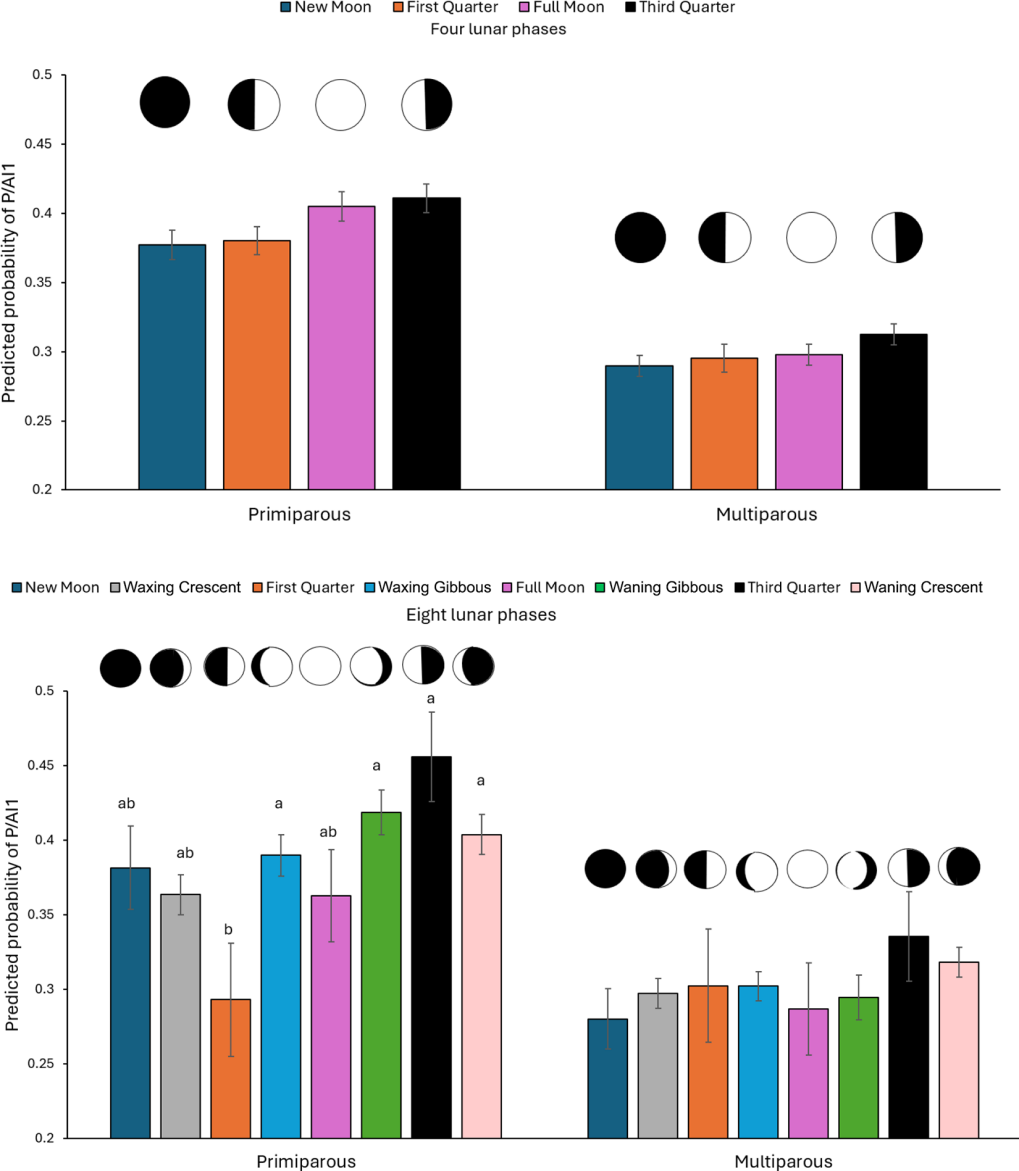
Predicted probabilities (LSM and SEM bars) for pregnancy at first AI (P/AI1) by lunar phase category. The lunar cycle was divided into 4 (top panel) and 8 (bottom panel) categories. The covariables used in the models included season of AI and DIM at AI. Cow ID was added as a random effect into the models. Interactions tested were not significant and were removed from the models. Different letters indicate a significant difference at *P* < 0.05. Two categorizations of the lunar cycle were used for the analysis: LP4 with 4 categories of equal duration (new moon to first quarter, first quarter to full moon, full moon to third quarter, and third quarter to new moon) and LP8 with 8 categories (new moon, first quarter, full moon, third quarter, set to 1 d duration; and waxing crescent, waxing gibbous, waning gibbous, and waning crescent, with 4 d of duration).

The resulting *P*-values from the logistic regression analysis for the main effect of the variable lunar cycle in primiparous and multiparous in LP4 were 0.006 and 0.07, respectively. Values in primiparous and multiparous in LP8 were <0.001 and 0.30, respectively. Interestingly, the logistic regression analyses in LP4 using the new moon category as reference identified greater odds of P/AI1 during the third quarter than during the new moon (odds ratio, **OR** [95% CI] = 1.15 [1.02–1.30] and 1.12 [1.01–1.24] for primiparous and multiparous cows, respectively). When LP8 was considered for the logistic analysis, associations between the lunar cycle and P/AI1 were only identified in primiparous cows for the first quarter compared with the new moon (OR [95% CI] = 0.67 [0.47–0.94]) (Table 1).

**Table 1:** Adjusted odds ratios (OR; 95% CI) for pregnancy at first insemination by category of lunar phase in lunar phase 4 (LP4) and lunar phase 8 (LP8) categorizations,^1^ considering new moon as the reference category^2^

Lunar phase	Primiparous			Multiparous		
	OR	95% CI	*P*-value	OR	95% CI	*P*-value
Four phases (LP4)						
New moon		Referent			Referent	
First quarter	1.01	0.90–1.15	0.83	1.03	0.93–1.14	0.60
Full moon	1.13	1.00–1.27	0.06	1.04	0.94–1.15	0.46
Third quarter	1.15	1.02–1.30	0.02	1.12	1.01–1.24	0.03
Eight phases (LP8)						
New moon		Referent			Referent	
Waxing crescent	0.93	0.71–1.29	0.55	1.09	0.87–1.36	0.57
First quarter	0.67	0.47–0.94	0.02	1.15	0.85–1.47	0.43
Waxing gibbous	1.03	0.78–1.35	0.79	1.11	0.89–1.39	0.49
Full moon	0.92	0.66–1.29	0.63	1.03	0.78–1.37	0.80
Waning gibbous	1.17	0.90–1.52	0.23	1.07	0.86–1.34	0.36
Third quarter	1.37	0.99–1.89	0.05	1.31	1.00–1.72	0.05
Waning crescent	1.10	0.85–1.42	0.47	1.21	0.97–1.50	0.18

1 Two categorizations of the lunar cycle were used for the analysis: LP4 with 4 categories of equal duration (new moon to first quarter, first quarter to full moon, full moon to third quarter, and third quarter to new moon) and LP8 with 8 categories (new moon, first quarter, full moon, third quarter, set to 1 d duration; and waxing crescent, waxing gibbous, waning gibbous, and waning crescent, with 4 d of duration).

2 The covariables used in the models included season of AI and DIM at AI. Cow ID was added as a random effect into the models. Interactions tested were not significant and were removed from the models.

There is a popular belief that the moon phases influence animal behavior and some physiological aspects of reproduction (Korringa, 1947). Supporting this idea, some studies in humans have reported variable associations between the lunar cycle and the menstrual cycle, sleep quality, and spontaneous delivery (Cutler et al., 1987; Ochiai et al., 2012; Komada et al., 2021). Similar to findings reported in women (Martens et al., 1988; Arliss et al., 2005), Yonezawa et al. (2016) described in dairy cows a significant effect of the lunar cycle on the frequency of birth, with an increase in the number of deliveries around the full moon phase in multiparae, whereas a similar trend was not evident in nulliparae.

To explore the influence of the moon phases on reproductive variables, we investigated the association between the lunar cycle and the probability of P/AI1 in Holstein cows that were maintained in an organic-certified dairy. This population of cows was especially suited for this research, as organic certification prohibits the use of exogenous hormones for reproductive management. In consequence, no synchronization of ovulation was attempted in these cows and estrus was detected considering behavioral changes in the cows. Additionally, as organic certification requires that cows have continuous access to an outdoor dry lot, as well as access to grazing during part of the year, permanent exposure to the natural environment is granted.

In the current study we identified some small associations among P/AI1 and the lunar cycle. The logistic regression analysis in the LP4 model indicated greater odds of pregnancy for AI occurring in the third quarter as compared with AI occurring in the new moon. Interestingly, this was the case for both primiparous and multiparous cows. On the contrary, when the lunar cycle was divided into 8 phases (LP8 categorization), a significant (*P* = 0.02) reduction in the odds of P/AI1 was only identified in primiparous cows for AI occurring during first quarter compared with AI during new moon. Nonetheless, it should be noticed that, concurring with the findings in LP4, the odds of P/AI1 were very close to being significantly greater in the third quarter than in the new moon of LP8 for both primiparous and multiparous cows (*P* = 0.05).

In agreement with our results, a study completed by Popescu et al. (2017) in Holstein, Angus, and Limousin cows identified an association between conception rate and the lunar phases, with the lowest conception rates during new moon breedings. Similarly, Aguirre et al. (2021) reported associations between the lunar cycle and some reproductive traits of tropical crossbred cows managed in a pasture-based system. In that study, conception at first service was greater during waning (59.9%) than in the crescent phase (55.8%). When the lunar cycle was divided into 4 quarters, first-service conception rate was significantly lower in quarters 1 and 2 (new moon to full moon) than in quarters 3 and 4 (full moon to new moon).

It is plausible to speculate that variations in luminescence or gravitational forces associated with the cycles of the moon could be involved in the differences in P/AI1 that we report in this study. Changes in nocturnal secretion of melatonin, which is in control of seasonal breeding in photoperiodic animals, are associated with variations in luminescence through the lunar cycle (Cajochen et al., 2013; Tamura et al., 2014). Moreover, the melatonin-sensitive pars tuberalis of the adenohypophysis is linked to GnRH production that has a crucial role in reproductive physiology. Connected with the involvement of melatonin in the control of breeding in photoperiodic animals (Dardente and Simonneaux, 2022; López-Gatius, 2024), recent studies considering Holstein populations demonstrated that the ovarian function in cows was related to changes in day length, with decreasing day length being associated with greater multiple ovulation and pregnancy rates (López-Gatius et al., 2023; López-Gatius, 2024).

In contrast, Yonezawa et al. (2016) proposed that variations in lunar gravity may affect the release of reproductive hormones through changes in blood pressure that is positively correlated with gravity. Interestingly, if variations in gravitational forces during the lunar cycle result in changes in blood pressure (Widjaja et al., 2015), this could alter the release patterns of certain hormones, such as oxytocin, which is increased as a response to elevations in blood pressure (Jankowski et al., 2020).

The ANOVA completed in this study identified significant differences in the predicted probabilities of P/AI1 among lunar phases only in the LP8 categorization in primiparous cows. The fact that LP8 considered only 1 d for new moon, first quarter, full moon, and third quarter phases may explain in part the differences between the LP4 and LP8 models, as this strategy allowed us to better isolate these 4 phases from the other 4 intermediate stages in the moon cycle. In the idea of maintaining uniformity in the logistic regression analyses, new moon was considered as the reference category for all the comparisons. Unlike the findings from the logistic regression analysis in LP4, the P/AI1 levels during the new moon phase were not the lowest for primiparous cows in the LP8 categorization. As a result, because this category used as a reference was not an extreme category in the LP8 analysis of primiparous cows, some differences identified in the ANOVA were not apparent in the logistic regression analysis (Figure 1 and Table 1).

As this is a retrospective study, some limitations should be considered. Due to organic regulations, cow management varied across the year, with allowance to grazing during part of spring and summer. It could be anticipated that a potential effect of the lunar cycle may be exacerbated during the grazing period where cows may be more exposed to the natural environment. Nonetheless, when analyzing breedings occurring during the grazing period (data not presented here), trends were consistent with those of the overall analysis. A second limitation is our inability to isolate potential effects of the lunar cycle on estrus expression versus conception. With the available data it is not possible to determine if the behavior of the cows during estrus is associated with the lunar phases, allowing the system to better identify cows that are truly in heat or if there are unidentified factors affecting the probability of conception or subsequent embryo survival.

In conclusion, in this study we identified small associations between the lunar cycle and pregnancy at first insemination of Holstein cows maintained in an organic-certified herd. This effects could be relevant when groups of animals are subject to specific reproductive interventions, such as synchronization of ovulation or embryo transfer. Nonetheless, validating the associations described here and clarifying the biological basis of the reported differences in P/AI1 would require controlled studies in other cattle populations.
